# Effects of high flow nasal cannula on the coordination between swallowing and breathing in postextubation patients, a randomized crossover study

**DOI:** 10.1186/s13054-021-03786-0

**Published:** 2021-10-19

**Authors:** Pornpan Rattanajiajaroen, Napplika Kongpolprom

**Affiliations:** Division of Pulmonary and Critical Care Medicine, Department of Medicine, Faculty of Medicine, Chulalongkorn University, King Chulalongkorn Memorial Hospital, The Thai Red Cross Society, Bangkok, Thailand

**Keywords:** High flow nasal cannula, Swallowing and breathing coordination, Post extubation patients

## Abstract

**Background:**

Timing of swallows in relation to respiratory phases is associated with aspiration events. Oxygen therapy possibly affects the timing of swallows, which may alter airway protective mechanisms.

**Objectives:**

To compare the coordination between swallowing and respiration during water infusion in post-extubation patients using high flow nasal oxygen (HFNO) with the coordination in those using low flow nasal oxygen (LFNO).

**Methods:**

We conducted a randomized controlled crossover study in post-extubation patients. The patients extubated within 48 h were randomly assigned to two groups, namely, HFNO and LFNO. The eligible patients in each group received either HFNO with fraction of inspired oxygen (FiO_2_) 0.35, flow 50 L per minute (LPM), and temperature 34 °C or LFNO 5 LPM for 5 min. The coordination between swallowing and respiration was observed during continuous infusion of 10-ml water one minute three times. Respiratory phases and swallowing were monitored using electrocardiogram (EKG)-derived respiratory signals and submental electromyography (EMG), respectively. The swallowing frequency and timing of swallows in relation to respiratory phases were recorded. The coordination between swallowing and respiration was classified into 4 patterns, namely I, E, I-E, and E-I swallows. (I; inspiration and E; expiration) Subsequently, after a 5-min washout period, the patients were switched to the other type of oxygen therapy using the same procedure. The Wilcoxon Signed-Rank Test was used for statistical analysis.

**Results:**

A total of 22 patients with a mean age of 56 years were enrolled in the study. The major indication for invasive mechanical ventilation was pneumonia with a median duration of endotracheal intubation of 2.5 days. The median total swallowing numbers (three minutes) were 18.5 times in the HFNO period and 21 times in the LFNO period (*p* = NS). The most common swallowing pattern was E-swallow. The patients using HFNO had higher numbers of E-swallow pattern (74.3% in HFNO vs 67.6% in LFNO; *p* = 0.048) and lower numbers of I-swallow pattern (14.3% in HFNO vs 23.1% in LFNO; *p* = 0.044). The numbers of other swallowing patterns were not different between the 2 groups.

**Conclusions:**

Compared with LFNO, HFNO significantly increased the E-swallow and decreased the I-swallow in post-extubation patients. The findings indicated that HFNO might reduce a risk of aspiration during the post-extubation period.

*Clinical trial No.*: Thai clinical trial TCTR20200206004 Registered February 4, 2020. URL: http://www.clinicaltrials.in.th/index.php?tp=regtrials&menu=trialsearch&smenu=fulltext&task=search&task2=view1&id=5740.

## Background

High flow nasal oxygen therapy (HFNO) has several physiological advantages over standard low flow nasal oxygen therapy (LFNO). Positive end-expiratory pressure (PEEP), a constant fraction of inspired oxygen (FiO_2_), pharyngeal dead-space washout, and enhanced mucociliary clearance are all benefits of HFNO. This oxygen delivery device is becoming more used in clinical practice, particularly in patients who have been extubated [1].

In patients at high risk of extubation failure, HFNO was not inferior to noninvasive ventilation in preventing post-extubation respiratory failure and reintubation [2]. Furthermore, Hernandez et al. found that, as compared to LFNO, HFNO significantly reduced the rate of reintubation within 72 h in patients at low risk of extubation failure [3].

One of the major benefits of HFNO in post-extubation patients is that it allows them to eat and drink orally without interrupting their treatment. However, there is a lack of evidence supporting the safety of HFNO regarding the risk of aspiration during oral ingestion.

Because of conditions including post-extubation dysphagia, incoordination between swallowing and breathing, and feeding intolerance, the risk of aspiration increases during the post-extubation period.

Post-extubation dysphagia has a prevalence of 3 to 62 percent, and it shares many of the same risk factors as post-extubation respiratory failure, including advanced age, prolonged intubation, and preexisting congestive heart failure. Furthermore, aspiration pneumonia, prolonged hospitalization, increased medical care costs, and mortality are all associated with post-extubation dysphagia [4].

According to the coordination between swallowing and respiration, breathing ceases briefly during swallowing due to inhibition of respiration at neural control centers in the brainstem and closure of the upper airway [5]. Swallowing normally occurs during expiration in healthy individuals and breathing resumes with the continuation of expiration after swallowing. Exhale–swallow–exhale or E-swallow is the most common pattern of the swallowing-breathing interaction, followed by inhale–swallow–exhale, or I-E swallow, which is considered one of the airway protection mechanisms. In addition, the alteration of this coordination, specifically inhale–swallow–inhale or I-swallow and exhale–swallow–inhale or E-I swallow, also appears with the lower percentages in healthy adults.

From previous studies, the incidence of aspiration was associated with the increase in percentages of I and E-I swallows, which are common in the elderly and patients with cerebrovascular, Parkinson's, and chronic obstructive pulmonary diseases [5, 6]. Despite the fact that many studies have looked into changes in swallowing and breathing coordination in various populations, the evidence in the post-extubation patients remains limited.

Only a few studies have looked into how airway pressure affects swallowing and breathing coordination. During continuous water infusion, Samson et al. found that bronchopulmonary receptor stimulation by nasal continuous positive airway pressure (nCPAP) in lamps lowered the frequency of swallowing and changed the patterns of swallowing-breathing interaction. During continuous infusion under nCPAP, alterations in this coordination, particularly a decrease in swallowing during inspiration (I and E-I swallows), might reduce the risk of aspiration [7].

Hori et al. demonstrated the effect of bi-level positive airway pressure (BiPAP) on the coordination of respiration and swallowing in 22 healthy volunteers. When comparing the BiPAP group to the control and continuous positive airway pressure (CPAP) groups, they found that the rate of inspiration following swallow was higher in the BiPAP group [8].

Corley et al. showed that HFNO increased end-expiratory lung volume and airway pressure [9]. As a result, HFNO might stimulate the bronchopulmonary receptor, causing changes in the timing of swallowing in relation to respiratory phases.

Researchers have become increasingly interested in the effects of HFNO on swallowing function in recent years. However, there was only one study in the healthy population that looked at swallowing function while using HFNO. In healthy subjects, Sanuki et al. found that HFNO reduced the swallowing latency time. Nonetheless, the timing of swallowing during a one-minute continuous infusion of water was not different between the HFNO and control groups [10].

To our knowledge, no study had looked specifically at the effect of HFNO on the relationship between swallowing and breathing during the post-extubation period. This study aimed to compare the swallowing-breathing coordination during continuous water infusion between HFNO and LFNO therapy in post-extubation patients.

## Methods

### Study design

Our study was the prospective, randomized, interventional, 2 × 2 crossover study. We conducted the trial in the medical inpatient department and medical intensive care unit at King Chulalongkorn Memorial Hospital, Bangkok, Thailand between June 2019 and February 2020. The protocol was approved by the Institutional Review Board of the Faculty of Medicine, Chulalongkorn University. This study was funded by Ratchadapisek Sompoch Fund, Faculty of Medicine, Chulalongkorn University, the grant number RA63/018.

### Participants

We enrolled patients at the age of 18–80 years who had been intubated for more than 48 h and had been extubated within the previous 48 h, were able to maintain adequate oxygen saturation (SpO_2_ ≥ 95%) using a low flow oxygen cannula 1–5 L per minute (LPM), had stable vital signs, and had passed the modified swallowing test with a score of more than 3 points. We excluded patients who were uncooperative or refused to participate in the study, had an enteral feeding contraindication, cerebrovascular disease or muscle weakness, head and neck cancer, structural abnormalities or a history of surgery in the oral cavity or pharyngeal area, or a previous diagnosis of dysphagia, had skin lesions that interfered with submental electromyography (EMG) monitoring, or had an automatic implantable cardioverter defibrillator or permanent pacemaker.

The modified water swallowing test was used to screen the eligible patients, which consisted of the post-extubation patients swallowing 3-milliliter (mL) water and subsequently swallowing their saliva at least twice [11]. We observed difficulty swallowing, choking, and/or breathing changes, as well as wet hoarseness. As a conclusion, the worst swallowing activity was graded. Passing the test was determined by a score of greater than 3 points.

### Equipment and techniques

Our patients received HFNO (OptiflowTM, Fisher and Paykel healthcare). Two surface electrodes attached to the skin at a submental region were used to assess muscle activity (bilateral suprahyoid muscles) during swallowing. Respiratory signals derived from the electrocardiogram (EKG) and EMG of respiratory muscles, including the bilateral sternocleidomastoid muscles, the second intercostal muscles, and the diaphragm, were used to track the phases of respiration. A 50-mL syringe, an infusion pump, and a 42-inch extension tube were used to provide a continuous infusion of water. The extension tube's distal tip was placed on the retromolar gingiva. One pulmonologist and two physiotherapists were among the three investigators. To screen eligible individuals, one physiotherapist performed a modified water swallowing test. The swallowing test (a one-minute continuous infusion of water) was performed by a pulmonologist and another physiotherapist, who analyzed submental EMG and ECG-derived breathing signals.

### Study protocol and measurements

**Fig. 1:**
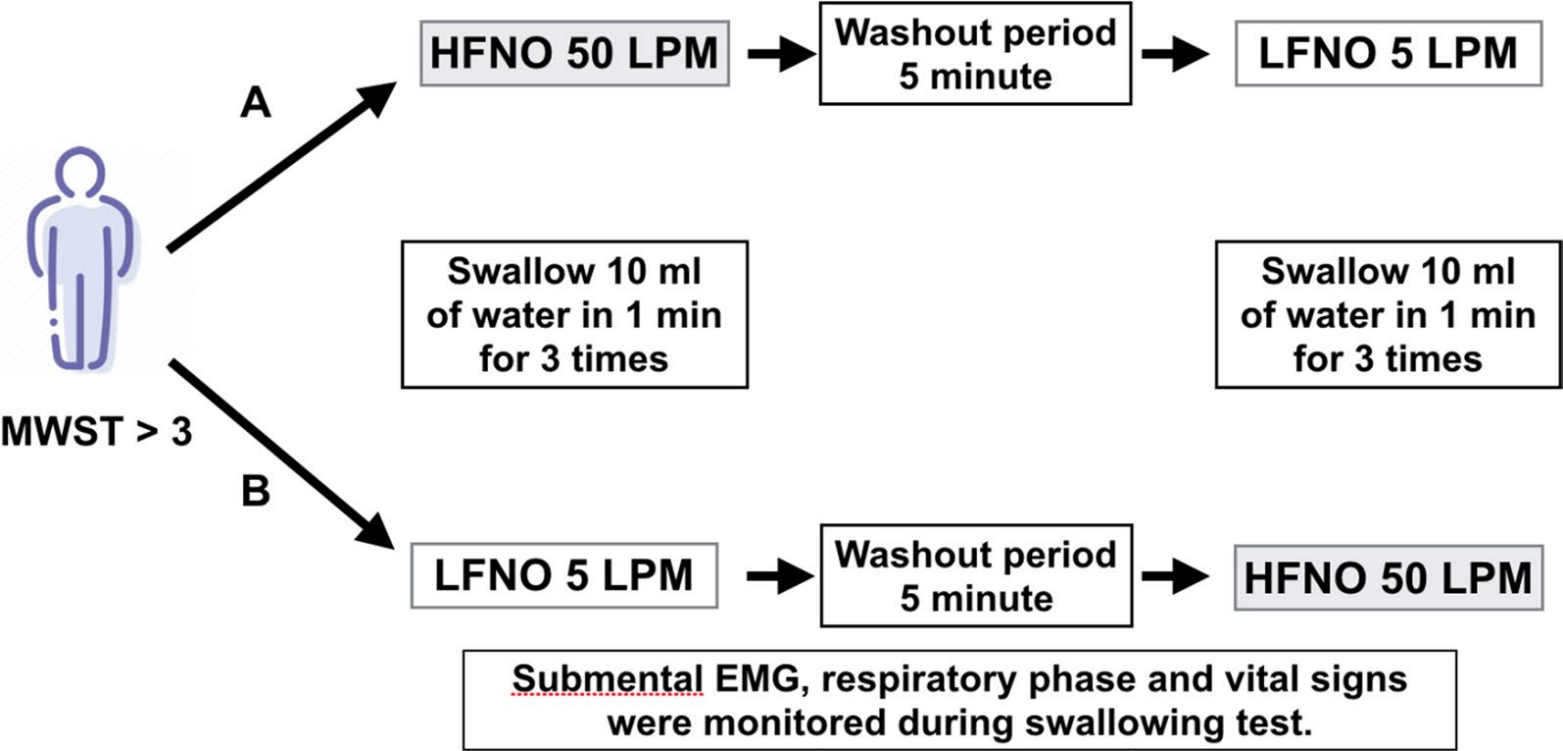
The study protocol. MWST, modified water swallowing test; EMG, electromyography; HFNO, high flow nasal oxygen; LFNO, low flow nasal oxygen; ml, milliliters; min, minute; LPM, liters per minute

Following informed consent, eligible patients were randomly allocated to one of two groups using a block of four randomization method. The patients in each group were in an upright position and were given either HFNO or LFNO for 5 min. The HFNO setting was a flow rate of 50 LPM, a temperature of 34 °C, and a FiO_2_ of 0.35, which could be adjusted to keep peripheral capillary oxygen saturation (SpO_2_) at least 95%, whereas the LFNO setting was a flow rate of 5 LPM to keep SpO_2_ at least 95%. The participants were then requested to swallow the 10-mL continuous water infusion in one minute. Three times, the continuous water swallowing test was performed. The number of swallows and the timing of swallowing in relation to respiratory phases, classified into four patterns, namely I; Inhale-swallow-inhale, E; Exhale-swallow-Exhale, I-E; Inhale-swallow-Exhale, and E-I; Exhale-swallow-Inhale, were recorded. ECG-derived respiratory signals and EMG of respiratory muscles were used to track the phases of respiration. The patient's blood pressure, heart rate, and oxygen saturation were all monitored. During the 5-min washout period after the first phase, LFNO with an adjustable flow rate of 1–5 LPM was given to achieve a SpO_2_ of at least 95%. The second period began after that. The patients were switched to the different type of oxygen therapy, and the procedure was repeated. (Fig. 1) If signs of aspiration appeared during the swallowing test, such as coughing, choking, dyspnea, a reduction in SpO_2_ of more than 2%, or vital sign abnormalities, the test would be promptly terminated and the patients would be rescued using an aspiration treatment protocol.

J. E. Ritchie et al. measured delivered FiO_2_ and airway pressures in healthy volunteers to assess the performance of a humidified nasal high flow system [12]. They began measuring hypopharyngeal pressure after a time of stabilization, assuring at least four breaths of stable capnography and oxygraphy data. To establish a steady-state, measurements were repeated at 1-min intervals. As a result, in our study, a 5-min HFNO application was expected to achieve the required airway pressure while also allowing patients to get acclimated to the equipment.

### Endpoints

The primary endpoint was the effects of HFNO on the coordination between swallowing and breathing in the post-extubation patients, as compared to LFNO.

The secondary endpoints included characteristics such as age, sex, comorbidities, and intubation duration that could affect the synchronization between swallowing and breathing in post-extubation patients.

### Statistical analysis

Sanuki et al. found that HFNO had some effects on swallowing-breathing coordination, although the results were not statistically significant [10]. The standard deviation (SD) for the data in the previous study was not available. To compute the sample size, we estimated the effect size to be 0.6 (moderate). The calculated sample size at a 0.05 level of significance was 22, which provided 80% power [13].

Continuous data were reported as mean ± SD or median [quartile1, quartile3]. Numbers or percentages were used to express the categorical data. The Chi-square, independent t-test, and Wilcoxon signed-rank tests were used to compare the baseline characteristics of the patients receiving HFNO and LFNO. The percentages of each swallowing pattern to the total number of swallows were used to represent the patterns of the relationship between swallowing and breathing (using mean values of three swallowing tests during the HFNO and LFNO periods). Wilcoxon signed-rank test was used to compare the percentage differences between the HFNO and LFNO periods. The factors that might alter the coordination between swallowing and respiration were determined by using Chi-square and independent t-tests or Wilcoxon signed-rank test. A median split was used on the percentages of each swallowing pattern to the total swallowing numbers to turn them into dichotomous variables, namely a low group and a high group [14]. Characteristics possibly associated with a high E-swallow group (patients with an E-swallowing percentage greater than the median value) were analyzed. A p-value of less than 0.05 was set as statistically significant.

## Results

**Fig. 2:**
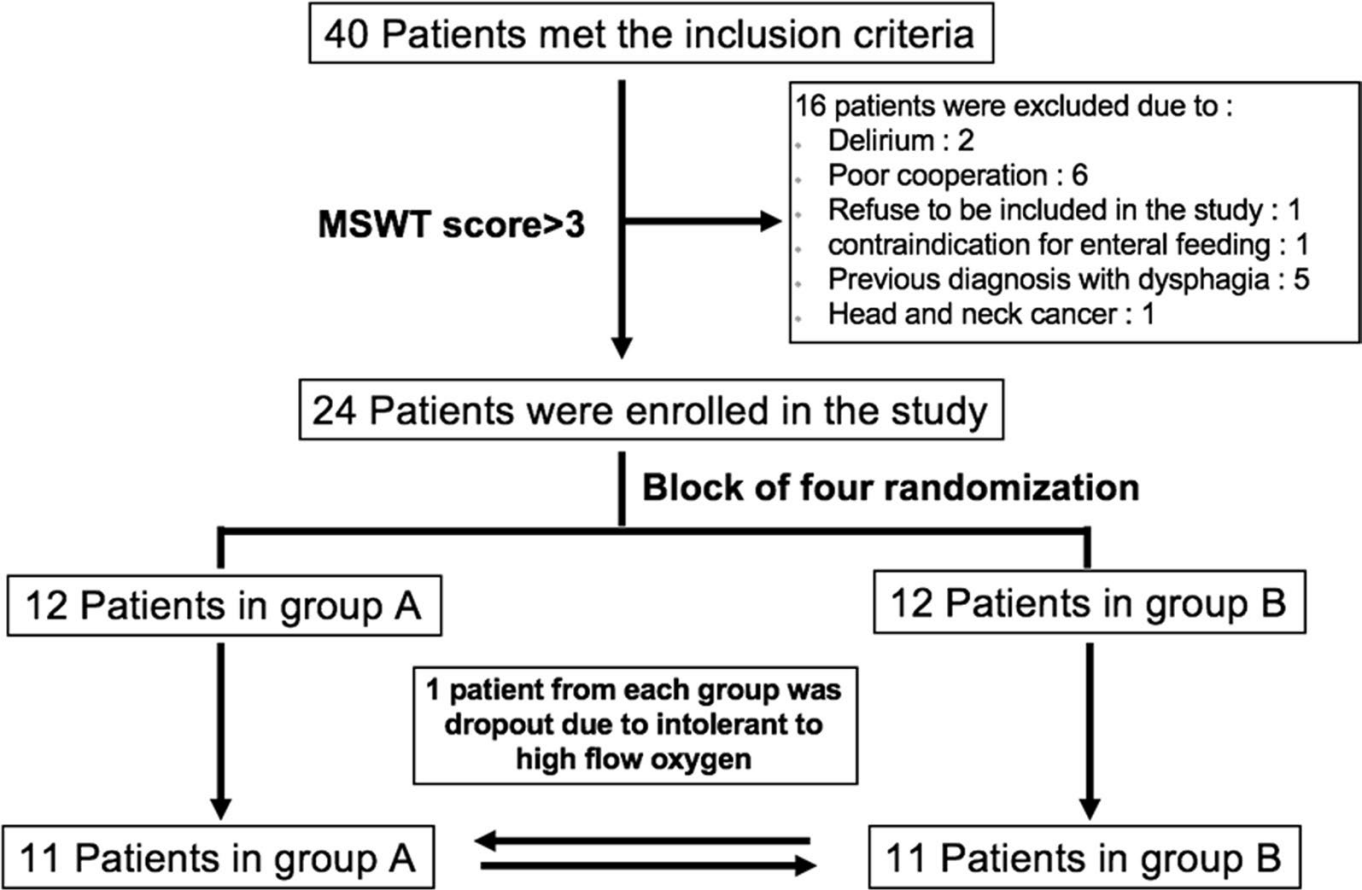
Flow of participants

A total of 40 patients met the inclusion criteria, however, 16 were excluded due to delirium, poor cooperation, enteral feeding contraindications, head and neck malignancy, prior dysphagia diagnosis, and refusal to participate in the study. Our study involved 24 patients (16 males and 8 females) who were assigned to two groups. (Fig. 2) Due to intolerance to HFNO, one patient from each group dropped out during the test. As a result, 22 patients completed the study. Table 1 shows the baseline characteristics. The average age of the eligible patients was 56 ± 12 years. The majority of them had hypertension (50%) or diabetes mellitus (40.9%). Pneumonia was the most common cause of invasive mechanical ventilation, followed by congestive heart failure, with a median duration of endotracheal intubation of 2.5 days. On the study date, the median APACHE II score was 6.

**Table 1:** Baseline characteristics of the study population

Baseline characteristics of study population (*n* = 22)	
Age (years), mean ± SD	56 ± 12
Sex: male, *n* (%)	15 (68.2%)
BMI (kg/m^2^), mean ± SD	22.3 ± 4.4
Indication for mechanical ventilation, * n* (%)	
Pneumonia	9 (41%)
Congestive heart failure	4 (18%)
Alteration of consciousness	4 (18%)
Lactic acidosis	3 (14%)
Asthmatic attack	1 (4.5%)
COPD with acute exacerbation	1 (4.5%)
APACHE II score on study date, median [Q1,Q3]	6 [4,7.75]
Endotracheal intubation (days), median [Q1,Q3]	2.5 [2,5.75]
Mechanical ventilation duration (days), median [Q1,Q3]	2.5 [2,5.5]
Comorbidities, * n* (%)	
Hypertension	11 (50%)
DM type 2	9 (40.9%)
Dyslipidemia	7 (31.8%)
Chronic kidney disease	6 (27.3%)
Ischemic heart disease	1 (4.5%)
History of sedative drugs use, * n* (%)	
Fentanyl	11 (50%)
Midazolam	4 (18.2%)
Propofol	1 (4.5%)
None	6 (27%)
History of neuromuscular blockade use, *n* (%)	1 (4.5%)

In the HFNO period, the median total swallowing number (three minutes) was 18.5 times and in the LFNO period, it was 21 times (*p* = 0.158). During the swallowing test, the mean expiratory time in one minute was significantly longer in the HFNO group (41.5 ± 4.0 s in HFNO vs 39.2 ± 2.9 s in LFNO, *p* < 0.001). However, there was no difference in the respiratory rate (19.5 [17, 21] rates per minute (rpm) in HFNO vs 20 [18, 24] rpm in LFNO, *p* = 0.068) during the swallowing test (Table 2).

**Table 2:** Primary outcome

Outcomes	Total patients (*n* = 22)		*p* value
	HFNO	LFNO	
Swallowing-breathing coordination, median [Q1,Q3]			
Total swallowing numbers**	18.5 [15, 22]	21 [17, 24]	0.158
I swallow (number)	2.5 [1, 4]	4 [3, 6]	0.002*
I swallow (%)	14.4 [6.7, 22.2]	23.1 [10.7, 28.5]	0.044*
E swallow (number)	14 [9, 21]	13.5 [11, 19]	0.452
E swallow (%)	74.3 [65.9, 86.7]	67.6 [55.6, 81]	0.048*
I-E swallow (number)	0.5 [0, 2]	1 [0, 2]	0.292
I-E swallow (%)	1.1 [0, 8.3]	6.1 [0, 9.3]	0.384
E-I swallow (number)	1 [0, 2]	1 [0, 2]	0.886
E-I swallow (%)	7.5 [0, 10.5]	4.5 [0, 9.5]	0.943

*p < 0.05; **Total swallowing numbers = summation of three swallowing tests; I swallow, inhale–swallow–inhale; E swallow, exhale–swallow–exhale; I-E swallow, inhale–swallow–exhale; E-I swallow, exhale–swallow–inhale

We calculated each swallowing pattern as a percentage of total swallows for determining swallowing-breathing synchronization. The E-swallow was the most common swallowing pattern in both HFNO and LFNO periods, followed by the I-swallow.

**Fig. 3:**
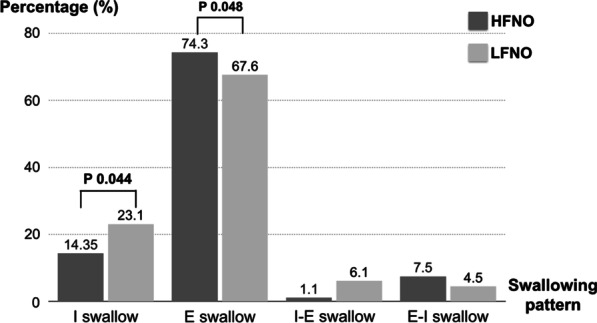
Comparison of percentages of each swallowing pattern to total swallows between the HFNO vs LFNO periods

**Fig. 4:**
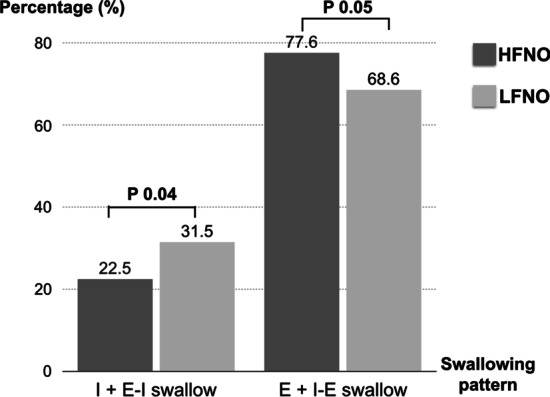
The summation of two unfavourable patterns (I and E-I swallows) and two favourable patterns (E and I-E swallows), compared between the HFNO vs LFNO periods

The patients using HFNO had a higher percentage of the E-swallow pattern (74.3% in HFNO vs 67.6% in LFNO; *p* = 0.048) and a lower percentage of the I-swallow pattern (14.3% in HFNO vs 23.1% in LFNO; *p* = 0.044). (Fig. 3) There was no difference in the number of other swallowing patterns between the two groups. When applying HFNO, we found that the favorable swallowing patterns (E and I-E swallows) were more common than the unfavorable swallowing patterns (I and E-I swallows). (Fig. 4).

Using the median values of the percentage of each swallowing pattern as cut-off values to categorize the patients into two subgroups and analyzing parameters possibly associated with the presence of each swallowing pattern, we determined the factors that might affect swallowing-breathing coordination for the secondary outcomes.

The percentage of E-swallows was defined by the percentage of swallows during the expiratory phase (Exhale–swallow–Exhale) divided by total swallows. The median percentage of E-swallows was 68%. By performing a median split test, patients with the E-swallow percentage of 68% or less were classified as a low E-swallow group, whereas patients with the E-swallow percentage of greater than 68% were classified as a high E-swallow group. During treatment with HFNO and LFNO, the high E-swallow group was older (mean 62 ± 11 vs 50 ± 11 years; *p* = 0.020), had a higher body mass index (BMI) (mean 24.2 ± 5.2 vs 20.4 ± 2.4 kg/m^2^; *p* = 0.045), and had hypertension (8 vs 3 patients; *p* = 0.033). Additionally, during HFNO treatment, the high E-swallow group had a higher BMI (mean 23.9 ± 4.7 vs 20.0 ± 2.7 kg/m^2^; *p* = 0.039). Nevertheless, no characteristic differences were identified between the low and high E-swallow groups during LFNO treatment (Table 3).

**Table 3:** Secondary outcomes; a comparison of characteristics between the low and high E-swallow groups, as defined by a median split on the percentage of E-swallowing during high flow oxygen therapy (HFNO), low flow oxygen therapy (LFNO), and both treatments

Variables	Total (*n* = 22)		*p* value	HFNO (*n* = 22)		*p* value	LFNO (*n* = 22)		*p* value
	E ≤ 68% (*n* = 11)	E > 68% (*n* = 11)		E ≤ 68% (*n* = 9)	E > 68% (*n* = 13)		E ≤ 68% (*n* = 11)	E > 68% (*n* = 11)	
Age (yr), mean ± SD	50 ± 11	62 ± 11	0.020*	50 ± 12	60 ± 11	0.054	55 ± 13	56 ± 12	0.647
Male, * n* (%)	8 (72.7%)	7 (63.6%)	0.647	6 (66.7%)	9 (69.2%)	0.899	8 (72.7%)	7 (63.6%)	0.647
Female, * n* (%)	3 (27.3%)	4 (36.4%)		3 (33.3%)	4 (30.8%)		3 (27.3%)	4 (36.4%)	
BMI (kg/m^2^), mean ± SD	20.4 ± 2.4	24.2 ± 5.2	0.045*	20.0 ± 2.7	23.9 ± 4.7	0.039*	21.6 ± 4.1	23.0 ± 4.8	0.476
Comorbidities, * n* (%)									
HT	3 (27.3%)	8 (72.7%)	0.033*	3 (33.3%)	8 (61.5%)	0.193	5 (45.5%)	6 (54.5%)	0.670
DM	3 (27.3%)	6 (54.5%)	0.193	3 (33.3%)	6 (46.2%)	0.548	4 (36.4%)	5 (45.5%)	0.665
DLP	3 (27.3%)	4 (36.4%)	0.647	3 (33.3%)	4 (30.8%)	0.899	3 (27.3%)	4 (36.4%)	0.647
CKD	3 (27.3%)	3 (27.3%)	1.000	3 (33.3%)	3 (23.1%)	0.595	4 (36.4%)	2 (18.2%)	0.338
IHD	0 (0%)	1 (9.1%)	0.306	0 (0%)	1 (7.7%)	0.394	0 (0%)	1 (9.1%)	0.306
APACHE II, mean ± SD	5.6 ± 2.7	6.6 ± 3.8	0.440	5.9 ± 2.7	6.2 ± 3.7	0.813	6.6 ± 3.4	5.6 ± 3.1	0.521
ETT duration (days),median	3 [2, 4]	2 2, 6]	0.972	4 [2, 6]	2 [2, 5]	0.268	3 [2, 4]	2 [2, 6]	0.972
Sedation, * n* (%)									
Fentanyl	6 (54.5%)	5 (45.5%)	0.670	6 (66.7%)	5 (38.5%)	0.193	6 (54.5%)	5 (45.5%)	0.670
Midazolam	3 (27.3%)	1 (9.1%)	0.269	3 (33.3%)	1 (7.7%)	0.125	2 (18.2%)	2 (18.2%)	1.000
Propofol	1 (9.1%)	0 (0%)	0.306	1 (11.1%)	0 (0%)	0.219	0 (0%)	1 (9.1%)	0.306

*p < 0.05; yr, year; BMI, body mass index; HT, hypertension; DM, diabetes mellitus; DLP, dyslipidemia; CKD, chronic kidney disease; IHD, ischemic heart disease; APACHE II, Acute Physiology And Chronic Health Evaluation II; ETT, endotracheal tube

## Discussion

The effect of HFNO on the synchronization of swallowing and breathing in post-extubation patients was demonstrated in our study. In comparison to the LFNO phase, the HFNO period had a higher percentage of the E-swallow pattern and a smaller percentage of the I-swallow pattern. Regardless of the type of oxygen therapy used, the E-swallow pattern was found to be the most common swallowing and breathing pattern in post-extubation patients. Furthermore, the presence of the E-swallow pattern was impacted by age, BMI, and hypertension.

During the post-extubation period, the use of HFNO improved the synchronization of swallowing and breathing. These findings could be explained by a number of factors.

To begin with, our research found that HFNO increased the likelihood of swallowing during the expiratory phase by lengthening the expiratory period. As a result, HFNO raised the E-swallow pattern significantly and breathing resume with expiration after swallowing protected the airways from aspiration.

Second, Thawanapong S and Kongpolprom N showed that HFNO reduced the mean-swallowing latency time in patients who had been extubated [15]. The period between swallowing onset (when the patients were requested to swallow) and the start of the first wave in the surface EMG was called the swallowing latency time. Aspiration was linked to a longer latency time, therefore the reduced latency time from HFNO could be due to more effective and coordinated swallowing [16]. However, due to the different techniques of the swallowing test, our study was unable to demonstrate the swallowing latency time.

Third, the subglottic pressure influenced swallowing efficiency by stabilizing the pharyngeal structure and stimulating airway mechanoreceptors [17]. Previous research has shown that lowering subglottic pressure lengthens the duration of pharyngeal contraction in healthy people [18]. Furthermore, the lower subglottic pressure slowed pharyngeal transit time in tracheostomized patients, potentially contributing to pharyngeal residue accumulation and aspiration [19, 20]. HFNO, on the other hand, produced positive airway pressure, which enhanced subglottic pressure and may have improved swallowing efficiency [21].

Finally, increasing end-expiratory lung volume resulting from positive airway pressure may trigger the Hering-Breuer reflex, which inhibits the following inhalation and reduces the I-swallow pattern [22].

However, the increased percentage of the E-swallow pattern in our study during the HFNO phase contradicted a previous study. Sanuki et al. found that the three varied flow rates (15, 30, and 45 LPM) of HFNO had no effect on swallowing-breathing coordination in healthy participants [10]. It might be explained by the different populations. Our research was conducted on post-extubation individuals who could benefit from HFNO physiology. Patients who had been extubated showed higher work of breathing, poorer respiratory mechanics, and a higher respiratory rate than healthy volunteers. HFNO was able to recruit alveoli and boost effective ventilation, reducing the work of breathing and respiratory rates during the post-extubation period [23]. Although there was no difference in respiratory rates between HFNO and LFNO in our study, improved respiratory physiology could lead to improved breathing comfort and swallowing facilitation. As a result, when using HFNO, the coordination of swallowing and breathing improved.

Furthermore, our study found that E-swallow was the most common swallowing-breathing pattern, followed by the I-swallow, whereas Sanuki et al. found that E-swallow was the most prevalent pattern, followed by the I-swallow-E in healthy volunteers [10]. The presence of the I-swallow was found to be higher in our study, which could be due to illness related to alterations in breathing patterns. It was supported by a previous study. In chronic obstructive pulmonary disease (COPD), Roxann Diez Gross et al. found an increase in the I-swallow, which possibly resulted from dynamic hyperinflation [24].

According to this study, there was a decrease in the percentage of the I-swallow pattern during the HFNO phase. Swallowing during inspiration has been shown to be a risk factor for aspiration pneumonia in several illnesses, including Parkinson's disease and COPD [25, 26]. We were unable to find a study that directly compared aspiration events between patients with higher and lower I-swallow patterns. Future research on the association between aspiration events and the different numbers of I-swallow is required.

Aging has been shown to alter the relationship between swallowing and respiration in previous research. In the elderly, there was a high occurrence rate of swallowing during inspiration. According to Bonnie Martin Harris et al. study, the mean age of the E-swallow in healthy adults was 56 years, while the mean age of the I-swallow and E-I swallow was 68 years [27]. In contrast, we demonstrated that the E-swallow appeared in a greater number in the more advanced age group. This finding could be due to a discrepancy in the study population and protocol. In our research, the degree of illness seemed to have a greater impact on swallowing coordination than age. In addition, we performed the continuous water swallowing test, while Bonnie Martin Harris et al. performed the bolus-swallowing test. Therefore, the result could not be directly compared.

Noticeably, our study demonstrated that patients with high E-swallow had a higher BMI and hypertension. To the best of our knowledge, no previous research has found a link between swallowing-breathing coordination and BMI or high blood pressure. Furthermore, we were unable to explain the findings by using swallowing and breathing physiology. As a result, additional research is required to confirm our findings.

Our results showed that HFNO improved swallowing-breathing coordination in post-extubation patients. These patients are one of the most common groups using HFNO. Therefore, the results may encourage clinicians to use this device with confidence, especially when patients begin to eat orally. However, future research should consider the aspiration event as one of the outcomes, as well as the long-term effects of HFNO use. It is also worth exploring the relationship between the percentages of each type of swallowing and aspiration incident.

There were some limitations in our study. The swallowing-breathing coordination was tested using a continuous water infusion, and the patients had to pass the modified swallowing test to be included in the study. As a result, our findings could not be applied to patients with dysphagia or patients who received a food bolus. Another limitation was that due to the different appearance of the oxygen devices, investigators and patients could not be blinded.

## Conclusions

This was the first study to show that providing HFNO instead of LFNO during the post-extubation period boosted E-swallow and decreased I-swallow. These findings suggest that HFNO may have some favorable effects on post-extubation patients' swallowing-breathing coordination. Future research is needed to determine whether HFNO reduces aspiration.

## Data Availability

All data generated or analyzed during this study are included in this published article and its supplementary information files.
